# Synthesis and carbonic anhydrase I, II, VII, and IX inhibition studies with a series of benzo[d]thiazole-5- and 6-sulfonamides

**DOI:** 10.1080/14756366.2017.1356295

**Published:** 2017-07-28

**Authors:** Morteza Abdoli, Andrea Angeli, Murat Bozdag, Fabrizio Carta, Ali Kakanejadifard, Hamid Saeidian, Claudiu T. Supuran

**Affiliations:** a Department of Chemistry, Faculty of Science, Lorestan University, Khorramabad, Iran;; b Dipartimento di Chimica, Laboratorio di Chimica Bioinorganica, Università degli Studi di Firenze, Sesto Fiorentino, Florence, Italy;; c Dipartimento Neurofarba, Sezione di Scienze Farmaceutiche, Polo Scientifico, Università degli Studi di Firenze, Sesto Fiorentino, Florence, Italy;; d Department of Science, Payame Noor University (PNU), Tehran, Iran

**Keywords:** Carbonic anhydrase, sulfonamide, inhibitor, benzo[d]thiazole, scaffold

## Abstract

A series of benzo[d]thiazole-5- and 6-sulfonamides has been synthesized and investigated for the inhibition of several human (h) carbonic anhydrase (CA, EC 4.2.1.1) isoforms, using ethoxzolamide (**EZA**) as lead molecule. 2-Amino-substituted, 2-acylamino- and halogenated (bromo-and iodo-derivatives at the heterocyclic ring) compounds led to several interesting inhibitors against the cytosolic hCA I, II and VII, as well as the transmembrane, tumor-associated hCA IX isoforms. Several subnanomolar/low nanomolar, isoform-selective sulfonamide inhibitors targeting hCA II, VII and IX were detected. The sharp structure–activity relationship for CA inhibition with this small series of derivatives, with important changes of activity observed even after minor changes in the scaffold or at the 2-amino moiety, make this class of scarcely investigated sulfonamides of particular interest for further investigations.

## Introduction

The carbonic anhydrases (CAs, EC 4.2.1.1) are a superfamily of metalloenzymes which catalyze the interconversion between CO_2_ and bicarbonate by using a metal hydroxide nucleophilic mechanism^1–9^. Seven distinct genetic CA families are known to date, the α-θ-CAs, which differ in their preference for metal ions used within the active site for performing the catalysis, their oligomerization state, but most importantly the three-dimensional fold of the protein^1–12^. In all cases, the apoenzymes are devoid of catalytic activity, the presence of the metal ion being essential both for catalysis as well as binding of inhibitors, many of which have biomedical applications^10–29^.

**Figure 1.**
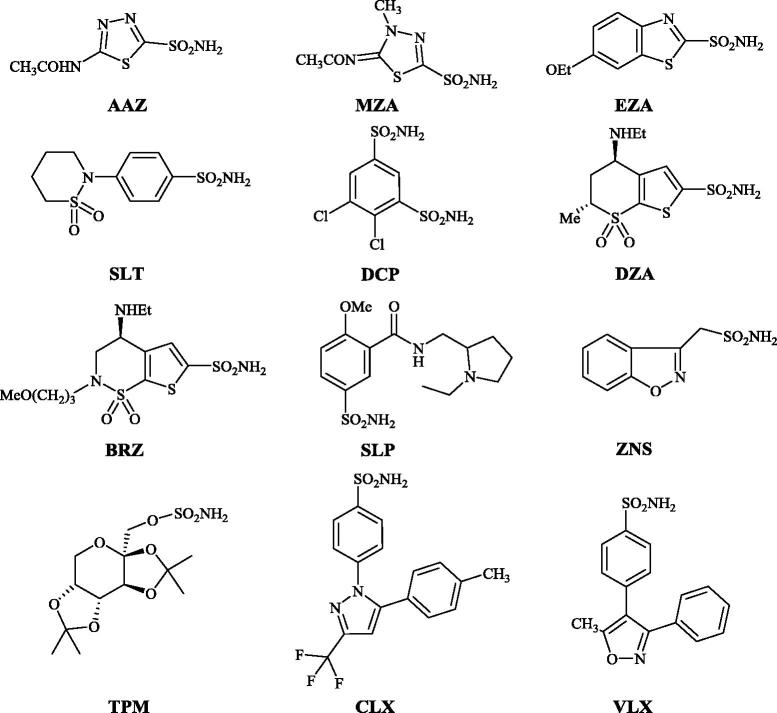
Clinically used CAIs of the sulfonamide and sulfamate type^10–19^.

Sulfonamides are the most important class of CA inhibitors (CAIs)^10–19^, with several compounds such as acetazolamide (**AAZ**), methazolamide (**MZA**), ethoxzolamide (**EZA**), sulthiame (**SLT**), dichlorophenamide (**DCP**), dorzolamide (**DZA**), brinzolamide (**BRZ**), sulpiride (**SLP**), zonisamide (**ZNS**), topiramate (**TPM**) (a sulfamate, not sulfonamide), celecoxib (**CLX**) and valdecoxib (**VLX**) (Figure 1).

Compounds **AAZ–VLX** may be considered as first/second generation CAIs. Their main problem is that they indiscriminately inhibit most of the human isoforms known to date^30–39^. Indeed, 16 such isozymes were described in non-primates, CA I**–**XV with two V-type isoforms, CA VA and CA VB, and 15 isoforms are known in primates, as CA XV is not expressed in these mammals^1–9^. The nonselective inhibition of most CA isoforms by the first/second generation sulfonamide CAIs is the reason why a large number of new such derivatives are constantly and permanently reported^20–39^.

## Materials and methods

### Chemistry

Anhydrous solvents and all reagents were purchased from Sigma-Aldrich, Alfa Aesar and TCI. All reactions involving air- or moisture-sensitive compounds were performed under a nitrogen atmosphere using dried glassware and syringes techniques to transfer solutions. Nuclear magnetic resonance (^1^H NMR, ^13^C NMR) spectra were recorded using a Bruker Avance III 400 MHz spectrometer in DMSO-d_6_. Chemical shifts are reported in parts per million (ppm), and the coupling constants (*J*) are expressed in Hertz (Hz). Splitting patterns are designated as follows: s, singlet; d, doublet; t, triplet; q, quadruplet; m, multiplet; brs, broad singlet; dd, double of doublets, and dt, double of triplets. The assignment of exchangeable protons (O*H* and N*H*) was confirmed by the addition of D_2_O. Analytical thin-layer chromatography (TLC) was carried out on Merck silica gel F-254 plates. Flash chromatography purifications were performed on Merck Silica gel 60 (230–400 mesh ASTM) as the stationary phase, and ethyl acetate (EtOAc)/*n*-hexane were used as eluents. Melting points (m.p.) were carried out in open capillary tubes and are uncorrected.

#### 4-Thioureido-benzenesulfonamide (1)

Sulfanilamide (2.0 g, 1.0 eq) was dissolved in a freshly prepared 3.5 M hydrochloric acid aqueous solution under gentle warming. The solution was cooled down to r.t. and potassium thiocyanate (1.0 eq) was added to reaction mixture then the mixture was heated at 90 °C for 3 h, cooled to r.t. to form precipitate which was filtered-off, washed with water, and dried under vacuum to afford the titled compound.

White solid, 93% yield; *δ*_H_ (400 MHz, DMSO-d_6_) 7.30 (2H, s), 7.70 (2H, d, *J* 8.8), 7.77 (2H, d, *J* 8.8), 10.01 (1H, s, exchange with D_2_O, N*H*); *δ*_C_ (100 MHz, DMSO-d_6_) 122.7, 127.2, 139.8, 143.4, 182.2; *m/z* (ESI positive) 232.01 [M + H]^+^. Experimental in agreement with reported data^40^.

#### 3-Thioureidobenzenesulfonamide (3)

3-Aminobenzensulfonamide (5.0 g, 1 eq) was dissolved in a freshly prepared 3.5 M hydrochloric acid aqueous solution by gentle warming, followed by treatment with potassium thiocyanate (1.0 eq) at r.t. and then heated to 90 °C for 12 h. The reaction mixture was cooled-down to r.t. and extracted with EtOAc (3 × 5.0 ml). The combined organic layers were washed with H_2_O (3 × 5.0 ml), dried over Na_2_SO_4_, filtered and concentrated to obtain a residue that was purified by silica gel column chromatography eluting with EtOAc/*n*-Hexane 70% v/v, followed by trituration with dichloromethane (DCM) to afford the titled compound.

White solid, 47% yield; *δ*_H_ (400 MHz, DMSO-d_6_) 7.39 (2H, s, exchange with D_2_O, SO_2_N*H*_2_), 7.56 (2H, m), 7.74 (1H, dt, *J* 1.8, 7.8), 7.96 (1H, d, *J* 1.8), 9.97 (1H, s, exchange with D_2_O, N*H*); *δ*_C_ (100 MHz, DMSO-d_6_) 120.5, 122.0, 126.7, 130.0, 140.8, 145.2, 182.4; *m/z* (ESI positive) 232.0 [M + H]^+^. Experimental in agreement with reported data^41^.

#### 2-Aminobenzo[d]thiazole-6-sulfonamide (2)

A suspension of 4-thioureido-benzenesulfonamide **1** (1.0 mmol, 1.0 eq) in CHCl_3_ (4.0 ml) was treated with Br_2_ (1.5 eq) drop-wise. The mixture was heated to 70 °C for 4.5 h, cooled-down to r.t. and the solvents were removed under reduced pressure to give a solid that was dissolved in H_2_O (5.0 ml). The aqueous solution was treated with NH_4_OH and stirred at 90 °C for 1 h. The formed precipitate was filtered-off, washed with H_2_O and dried under vacuum to afford the titled compound.

White solid, 80% yield; *δ*_H_ (400 MHz, DMSO-d_6_) 7.23 (2H, s, exchange with D_2_O, SO_2_N*H*_2_), 7.45 (1H, d, *J* 8.4), 7.69 (1H, dd, *J* 8.4, 1.8), 7.89 (2H, s, exchange with D_2_O, N*H*_2_), 8.15 (1H, d, *J* 1.8); *δ*_C_ (100 MHz, DMSO-d_6_) 118.0, 120.1, 124.6, 131.8, 137.2, 156.0, 170.3; *m/z* (ESI positive) 230.00 [M + H]^+^. Experimental in agreement with reported data^41^.

#### 2-Aminobenzo[d]thiazole-5-sulfonamide (4)

A suspension of **3** (1.2 g, 1.0 eq.) in CHCl_3_ (15.0 ml) was treated with Br_2_ (1.5 eq) in CHCl_3_ (1.0 ml) drop-wise. The mixture was heated to 70 °C for 12 h, cooled down to r.t., the solvent eliminated in vacuum to give a residue that was dissolved in H_2_O (5.0 ml) and treated with NH_4_OH, followed by 1 h stirring at 90 °C. The cooled reaction mixture was filtered, washed with water and dried under vacuum to afford the titled compound.

White solid, 45% yield; *δ*_H_ (400 MHz, DMSO-d_6_) 7.42 (1H, t, *J* 8.0), 7.49–7.56 (4H, m, 2H exchange with D_2_O, SO_2_N*H*_2_), 7.69 (2H, s, exchange with D_2_O, N*H*_2_); *δ*_C_ (100 MHz, DMSO-d_6_) 120.39, 121.4, 126.7, 128.5, 137.7, 155.3, 169.7; *m/z* (ESI positive) 230.00 [M + H]^+^.

#### 2-Amino-4-bromobenzo[d]thiazole-6-sulfonamide (5)

A suspension of **2** (0.75 g, 1 eq) in chloroform (15.0 ml) was treated with a solution of Br_2_ (8.0 eq) in chloroform (2.5 ml) drop-wise. The mixture was heated to 70 °C for 4 h. After cooling to r.t. the solvents were removed under reduced pressure. The obtained solid was dissolved in water (5.0 ml) and treated with ammonium hydroxide (pH =10), then the reaction mixture stirred for 1 h at 90 °C. The precipitated solid was filtered under vacuum, washed with H_2_O (3 × 5.0 ml), then with *n*-Hexane (3 × 3.0 ml) and dried to afford the titled compound.

Orange solid, 68% yield; *δ*_H_ (400 MHz, DMSO-d_6_) 7.35 (2H, s, exchange with D_2_O, SO_2_N*H*_2_), 7.89 (1H, s), 8.17 (1H, s), 8.26 (2H, s, exchange with D_2_O, N*H*_2_); *δ*_C_ (100 MHz, DMSO-d_6_) 110.7, 119.7, 127.6, 132.6, 138.1, 154.5, 170.9; *m/z* (ESI positive) 307.9 [M + H]^+^.

#### 2*-Amino-4-bromobenzo[d]thiazole-5-sulfonamide (6)*

A suspension of **4** (0.2 g, 1.0 eq) in chloroform (4.0 ml) was treated with a solution of Br_2_ (6.0 eq) in chloroform (1.0 ml) drop-wise. The mixture was heated to 70 °C for 12 h. After cooling to r.t. the solvents were removed under reduced pressure. The obtained solid was dissolved in water (5.0 ml) and treated with ammonium hydroxide (pH =10), then the reaction mixture stirred for 1 h at 90 °C. After cooling, the reaction mixture was extracted with EtOAc (3 × 5 ml). The combined organic layers were washed with H_2_O (3 × 5.0 ml), dried over Na_2_SO_4_, filtered and concentrated to obtain a residue that was purified by silica gel column chromatography eluting with EtOAc/*n*-Hexane 70% v/v to afford the titled compound.

Orange solid, 19% yield; *δ*_H_ (400 MHz, DMSO-d_6_) 7.40 (1H, d, *J* 8.4), 7.66 (2H, s, exchange with D_2_O, SO_2_N*H*_2_), 7.69 (1H, d, *J* 8.4), 8.08 (2H, s, exchange with D_2_O, N*H*_2_); *δ*_C_ (100 MHz, DMSO-d_6_) 114.3, 120.6, 128.8, 129.8, 137.1, 152.9, 170.1; *m/z* (ESI negative) 305.7 [M-H]^−^.

#### 2-Amino-4-iodobenzo[d]thiazole-6-sulfonamide (7)

A solution of **2** (0.3 g, 1.0 eq) in methanol (3.0 ml) was treated with iodine monochloride (4.0 eq) in methanol (1.0 ml) drop-wise. The mixture was heated to reflux temperature for 12 h. After cooling to room temperature, the reaction mixture was extracted with EtOAc (3 × 5.0 ml). The combined organic layers were washed with H_2_O (3 × 5.0 ml), dried over Na_2_SO_4_, filtered and concentrated to obtain a residue that was purified by silica gel column chromatography eluting with EtOAc/*n*-Hexane 70% v/v to afford the titled compound.

Dark orange solid, 31% yield; *δ*_H_ (400 MHz, DMSO-d_6_) 7.31 (2H, s, exchange with D_2_O, SO_2_N*H*_2_), 8.06 (1H, d, *J* 2.0), 8.16 (1H, d, *J* 2.0), 8.21 (2H, s, exchange with D_2_O, N*H*_2_); *δ*_C_ (100 MHz, DMSO-d_6_) 84.4, 119.9, 129.9, 133.1, 138.3, 157.0, 169.7; *m/z* (ESI positive) 355.9 [M + H]^+^.

#### 2-Amino-4-iodobenzo[d]thiazole-5-sulfonamide (8)

A solution of **4** (0.2 g, 1.0 eq) in methanol (3.0 ml) was treated with a solution of iodine monochloride (4.0 eq) in methanol (1.0 ml) drop-wise. The mixture was heated to reflux temperature for 12 h. After cooling to room temperature, the reaction mixture was extracted with EtOAc (3 × 5 ml). The combined organic layers were washed with H_2_O (3 × 5.0 ml), dried over Na_2_SO_4_, filtered and concentrated to obtain a residue that was purified by silica gel column chromatography eluting with EtOAc/*n*-Hexane 70% v/v to afford the titled compound.

Dark orange solid, 16% yield; *δ*_H_ (400 MHz, DMSO-d_6_) 7.25 (1H, d, *J* 8.0), 7.63 (2H, s, exchange with D_2_O, SO_2_N*H*_2_), 7.87 (1H, d, *J* 8.0), 8.03 (2H, s, exchange with D_2_O, N*H*_2_); *δ*_C_ (100 MHz, DMSO-d_6_) 84.5, 119.9, 129.9, 133.2, 138.3, 157.0, 169.7; *m/z* (ESI positive) 355.8 [M + H]^+^.

#### N-(6-sulfamoylbenzo[d]thiazol-2-yl)acetamide (9)

A solution of **2** (1.0 g, 1.0 eq) in acetic acid (2.0 ml) was cooled to 0 °C followed by drop-wise addition of acetic anhydride (1.2 eq). The reaction mixture was refluxed for 3 h then excess of solvents were removed under reduced pressure to obtain a residue which was washed with Et_2_O (3 × 5 ml) to obtain titled compound.

White solid, 96% yield; *δ*_H_ (400 MHz, DMSO-d_6_) 2.27 (3H, s), 7.39 (2H, s, exchange with D_2_O, SO_2_N*H*_2_), 7.90 (2H, d, *J* 1.2), 8.50 (1H, t, *J* 1.2), 12.59 (1H, s, exchange with D_2_O, N*H*); *δ*_C_ (100 MHz, DMSO-d_6_) 23.7, 121.0, 121.4, 124.8, 132.4, 139.9, 151.7, 161.9, 170.7; *m/z* (ESI positive) 272.0 [M + H]^+^.

#### N-(4-bromo-6-sulfamoylbenzo[d]thiazol-2-yl)acetamide (10)

A solution of **5** (0.1 g, 1.0 eq) in acetic acid (0.5 ml) was cooled to 0 °C followed by drop-wise addition of acetic anhydride (1.2 eq) then the mixture was refluxed for 3 h. The excess of solvents was removed under reduced pressure. The obtained solid was treated with sodium bicarbonate (1 N, 3.0 ml), then the reaction mixture was extracted with EtOAc (3 × 5 ml). The combined organic layers were washed with H_2_O (3 × 5.0 ml), dried over Na_2_SO_4_, filtered and concentrated under reduced pressure to afford the titled compound.

Orange solid, 93% yield; *δ*_H_ (400 MHz, DMSO-d_6_) 2.24 (3H, s), 7.44 (2H, s, exchange with D_2_O, SO_2_N*H*_2_), 8.05 (1H, s), 8.50 (1H, s), 12.59 (1H, s, exchange with D_2_O, N*H*); *δ*_C_ (100 MHz, DMSO-d_6_) 23.6, 114.1, 120.5, 127.5, 133.2, 140.8, 149.8, 162.7, 171.0; *m/z* (ESI positive) 349.8 [M + H]^+^.

#### N-(5-sulfamoylbenzo[d]thiazol-2-yl)acetamide (11)

A solution of **4** (0.1 g, 1.0 eq) in acetic acid (0.5 ml) was cooled to 0 °C followed by drop-wise addition of acetic anhydride (1.2 eq) then the mixture was refluxed for 3 h. The excess of solvents was removed under reduced pressure. The obtained solid was treated with sodium bicarbonate (1 N, 3 ml), then the reaction mixture was extracted with EtOAc (3 × 5 ml). The combined organic layers were washed with H_2_O (3 × 5.0 ml), dried over Na_2_SO_4_, filtered and concentrated under reduced pressure to afford the titled compound.

White solid, 89% yield; *δ*_H_ (400 MHz, DMSO-d_6_) 2.27 (3H, s), 7.66 (1H, t, *J* 7.8), 7.71 (2H, s, exchange with D_2_O, SO_2_N*H*_2_), 8.80 (1H, dd, *J* 7.8, 1.0), 7.98 (1H, dd, *J* 7.8, 1.0), 12.45 (1H, s, exchange with D_2_O, N*H*); *δ*_C_ (100 MHz, DMSO-d_6_) 23.6, 122.7, 124.6, 127.1, 128.8, 138.6, 150.9, 161.5, 170.6; *m/z* (ESI positive) 272.0 [M + H]^+^.

#### 2-((6-Sulfamoylbenzo[d]thiazol-2-yl)carbamoyl)benzoic acid (12)

A solution of **2** (0.3 g, 1.0 eq) in dry DMF (3.0 ml) was treated with phthalic anhydride (1.0 eq), then the mixture was refluxed for 4 h. The reaction mixture was extracted with EtOAc (3 × 5.0 ml). The combined organic layers were washed with H_2_O (3 × 5.0 ml), dried over Na_2_SO_4_, filtered and concentrated under reduced pressure to afford the corresponding pure mixture of 2 isomers in (50:50) as evidenced by ^1^H NMR integration.

White solid, 100% yield; *δ*_H_ (400 MHz, DMSO-d_6_) 7.41 (2H, s, exchange with D_2_O, SO_2_N*H*_2_), 7.51 (2H, s, exchange with D_2_O, SO_2_N*H*_2_), 7.67–7.77 (4H, m), 7.93–8.03 (7H, m), 8.11–8.13 (2H, m), 8.22 (1H, d, *J* 8.4), 8.56 (1H, s, exchange with D_2_O, N*H*), 8.71 (1H, d, *J* 1.6, exchange with D_2_O, N*H*); *δ*_C_ (100 MHz, DMSO-d_6_) 121.0, 121.2, 121.5, 123.4, 124.8, 125.0, 125.1, 129.0, 130.6, 130.8, 131.3, 131.9, 132.5, 132.9, 133.2, 136.5, 137.0, 140.0, 141.5, 151.7, 151.8, 156.2, 162.1, 165.2, 167.8, 169.6; *m/z* (ESI positive) 377.9 [M + H]^+^.

### CA inhibition

An Applied Photophysics stopped-flow instrument has been used for assaying the CA catalyzed CO_2_ hydration activity^42^. Phenol red (at a concentration of 0.2 mM) has been used as indicator, working at the absorbance maximum of 557 nm, with 20 mM Hepes (pH 7.5) as buffer, and 20 mM Na_2_SO_4_ (for maintaining constant the ionic strength), following the initial rates of the CA-catalyzed CO_2_ hydration reaction for a period of 10–100 s. The CO_2_ concentrations ranged from 1.7 to 17 mM for the determination of the kinetic parameters and inhibition constants. For each inhibitor, at least six traces of the initial 5–10% of the reaction have been used for determining the initial velocity. The uncatalyzed rates were determined in the same manner and subtracted from the total observed rates. Stock solutions of inhibitor (0.1 mM) were prepared in distilled–deionized water and dilutions up to 0.01 nM were done thereafter with the assay buffer. Inhibitor and enzyme solutions were preincubated together at room temperature (15 min) prior to assay, in order to allow for the formation of the E–I complex. Data from Table 1 were obtained after 15 min incubation of enzyme and inhibitor, as for all sulfonamides reported earlier^43–50^. The inhibition constants were obtained by non-linear least-squares methods using PRISM 3 and the Cheng–Prusoff equation, as reported earlier^51–55^ and represent the mean from at least three different determinations. All CA isoforms were recombinant ones obtained in-house as reported earlier^51–60^.

**Table 1.** Inhibition data of human CA isoforms hCA I, II, VII and IX with compounds **1–12** in comparison with the standard sulfonamide inhibitors **AAZ** and **EZA** by a stopped flow CO_2_ hydrase assay^42^.

	K_I_ (nM)^a^			
Compound	hCA I	hCA II	hCA VII	hCA IX
**1**	470.8	70.0	75.4	32.9
**2**	84.1	33.6	84.2	3.7
**3**	917.1	149.2	75.0	295.6
**4**	795.2	369.0	56.5	38.2
**5**	305.7	8.7	31.1	16.2
**6**	704.8	7.8	0.8	29.6
**7**	606.2	15.1	92.3	212.0
**8**	481.6	51.5	42.2	100.0
**9**	361.2	20.8	54.4	23.2
**10**	2327	210.7	80.6	34.4
**11**	340.6	42.0	81.5	32.6
**12**	97.1	13.5	46.5	10.0
**AAZ**	250.0	12.1	5.7	25.8
**EZA**	25.0	8.1	0.8	34.2

a Mean from 3 different assays, by a stopped flow technique (errors were in the range of ±5–10% of the reported values).

## Results and discussion

### Chemistry

**Scheme 1.**
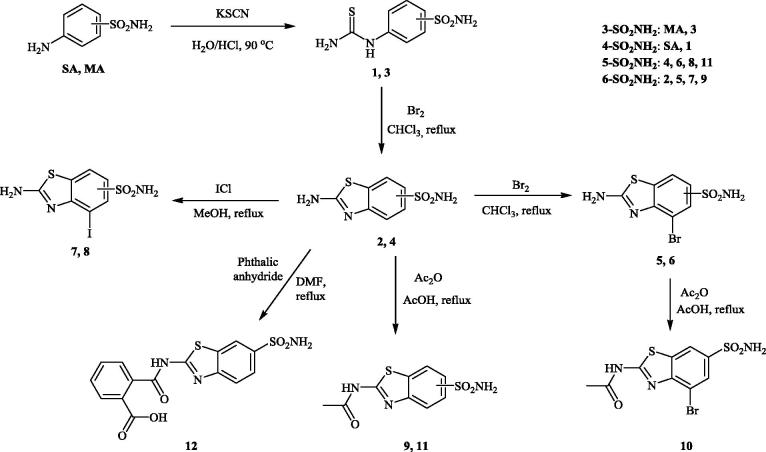
Preparation of sulfonamides **1–12** investigated in this article, starting from sulfanilamide SA or metanilamide MA.

Most of the CAIs generated in our group over the last two decades were designed by using the tail approach^15^^,^^32^^,^^33^. By choosing various functionalities that are appended on the scaffold of aromatic/heterocyclic sulfonamides in such a way as to interact with the middle and rim parts of the CA active site, a large number of isoform-selective CAIs were obtained^15–36^. Here on the other hand, we decided to explore a variant of the ring approach^1^^,^^4^, using ethoxzolamide (**EZA)** (Figure 1) as lead molecule. A series of benzothiazole-6-sulfonamides are reported here, which differ from EZA mainly by the position of the sulfamoyl moiety and by the presence of various substituents at the heterocyclic ring, in various positions (Scheme 1).

Sulfanilamide (SA)/metanilamide (MA) were reacted with potassium isocyanate in the presence of HCl, leading to the corresponding isothiocyanato-benzenesulfonamides **1** and **3**, respectively. Bromination of these key intermediates led to the ring closure and formation of the regiomeric benzothiazole sulfonamides **2** and **4**, respectively (Scheme 1). These compounds were acetylated and/or halogenated, leading to the small library of derivatives shown in Scheme 1 (see Experimental for details). Four of these derivatives have the sulfamoyl moiety in the 5 position of the benzothiazole ring, whereas the remaining ones in the 6 position (Scheme 1).

### Carbonic anhydrase inhibition

The synthesized compounds **1–12** were investigated for their inhibitory effects against four physiological relevant isoforms, i.e. hCA I, II, VII and IX, by means of a stopped flow CO_2_ hydrase assay^42^.

The following structure–activity relationship (SAR) can be drawn from data of Table 1:

- hCA I was inhibited by all these sulfonamides, with inhibition constants ranging between 84.1 and 2327 nM. Two compounds, **2** and **12**, had K_I_s < 100 nM, and they have both 2-amino-benzothiazole-6-sulfonamide derivatives. However **2** has no substituents on the amino functionality, whereas **12** has the bulky phthaloyl-monoamide functionality, proving thus that the SAR for inhibiting this isoform with sulfonamides investigated here is rather complex. Both these compounds were around three–four times less effective hCA I inhibitors compared to **EZA** (K_I_ of 25 nM). Introduction of halogens on the benzothiazole scaffold of acetylation of the amino group led to compounds with less effective hCA I inhibitory properties compared to **2**. The same was true for the compounds from the benzothiazole-5-sulfonamide series. For the simple derivatives, generally the 6-sulfamoyl derivatives were more effective CAIs compared to the corresponding 5-sulfamoyl ones (e.g. compare **2** and **4**) whereas for the halogeno-substituted ones the behavior was not so clear-cut, with the bromoderivatives **5** and **6** behaving like the parent aminoderivatives, whereas an opposite effect was observed for the iododerivatives **7** and **8**, case in which the 5-sulfonamide was a better inhibitor compared to the isomeric 6-sulfonamide (Table 1).
- hCA II was effectively inhibited by sulfonamides investigated here, with K_I_s in the range of 7.8–369 nM. The best inhibitors were **2, 5–9, 11** and **12**, with inhibition constants in the range of 7.8–51.5 nM. They belong to both the 5- as well as 6-sulfonamide series. The 2-amino-benzothiazole-6-sulfonamide derivative **2** was already an effective hCA II inhibitor, and its derivatization (acetylation and mono-phthaloylation) led to even better inhibitors (compare **9**, **12,** and **2**). Halogenation of **2** led to very effective inhibitors, with both the bromo-and iodo-derivatives **5**, **7**, having K_I_s of 8.7 and 15.1 nM, respectively. However, bromination of the acetylated derivative **9** led to a strong loss of inhibitory effects in the halogenated derivative **10**. For the 5-sulfonamide series, the situation was rather different. The parent compound, 2-amino-benzothiazole-5-sulfonamide derivative **4** was a modest hCA II inhibitor, with an inhibition constant of 369 nM. Its derivatization by introduction of halogeno atoms on the heterocyclic ring, as in **6** and **8**, or the acetylation of the amino moiety, as in **11**, led to a potent increase in the inhibitory power, with the bromo-derivative **6** being one of the best inhibitors on the series (K_I_ of 7.8 nM, being more effective than **AAZ** or **EZA**, see Table 1).
- Effective inhibition was observed also for the brain-associated, cytosolic isoform hCA VII, a recently validated target for neuropathic pain^61^^,^^62^. The sulfonamides investigated here showed K_I_s in the range of 0.8–92.3 nM. Most of these derivatives were in fact medium potency inhibitors, with inhibition constants of 42.2–92.3 nM, except **6** (K_I_ of 0.8 nM) and **5** (K_I_ of 31.1 nM). Both of them are the bromine derivatives of the isomeric 2-amino-benzothiazole-sulfonamides, with the 5-sulfonamide derivative **6** being 38.8 times a better hCA VII inhibitor compared to the 6-sulfonamide one **5** (Table 1). Compound **6** was equipotent to **EZA** for inhibiting this isoform.
- The tumor-associated, transmembrane isoform hCA IX was also effectively inhibited by these sulfonamides, with K_I_s in the range of 3.7–295.6 nM (Table 1). The most effective inhibitors were **2, 4–6**, and **9–12**, with K_I_s in the range of 3.7–38.2 nM, the same range as the clinically used, standard inhibitors **AAZ** and **EZA** (Table 1). By comparing the two amino derivatives **2** and **4**, it may be observed that in this case the 6-sulfonamide **2** was around 10 times a better hCA IX inhibitor compared to the isomeric 5-sulfonamide **4**. Halogenation of **2** generally led to a decrease of the inhibitory potency, whereas acylation of the amino group had the same effect (but the loss of potency was smaller). Rather similar effects were observed for the 5-sulfonamide series, except that the bromination led to a slight increase in the hCA IX inhibitory power (compare **4** and **6**).
- Some of the reported sulfonamides tended to show some selectivity for inhibiting one CA isoform over the remaining ones. Examples in this sense are **6**, which showed a good hCA VII selective inhibition profile, **9, 10, 11,** and **12**, which were effective hCA II and IX inhibitors, but weaker hCA I and VII inhibitors. However, these compounds possess a rather compact scaffold that probably binds deep within the CA active site, where most amino acid residues are conserved among the various isoforms. This is probably the reason why they show a rather low isoform-selective inhibition profile, a problem they share with most inhibitors of the first and second generation, which have been designed by the ring approach. As we stressed here and in other papers^1^^,^^2^^,^^5^, this issue has been resolved by the using tail approach, which led to many classes of isoform-selective CAIs^63–65^.

## Conclusions

A small series of benzo[d]thiazole-5- and 6-sulfonamides has been synthesized by following literature procedures, and investigated for the inhibition of several hCA isoforms, using ethoxzolamide as lead molecule. 2-Amino-substituted, 2-acylamino- and halogenated (bromo-and iodo-derivatives at the heterocyclic ring) compounds led to several interesting inhibitors against the cytosolic hCA I, II, and VII, as well as the transmembrane, tumor-associated hCA IX isoforms. Several subnanomolar/low nanomolar, isoform-selective sulfonamide inhibitors targeting hCA II, VII and IX were detected. The sharp structure–activity relationship for CA inhibition with this small series of derivatives, with important changes of activity observed even after minor changes in the scaffold or at the 2-amino moiety, make this class of scarcely investigated sulfonamides of particular interest for further investigations.
